# Cytokine responses of bovine macrophages to diverse clinical *Mycobacterium avium *subspecies *paratuberculosis *strains

**DOI:** 10.1186/1471-2180-6-10

**Published:** 2006-02-14

**Authors:** Harish K Janagama, Kwang il Jeong, Vivek Kapur, Paul Coussens, Srinand Sreevatsan

**Affiliations:** 1 Department of Veterinary Population Medicine, University of Minnesota, Saint Paul, MN, USA; 2 Food Animal Health Research Program, The Ohio State University, Wooster, OH, USA; 3 Biomedical Genomics Center and Department of Microbiology, University of Minnesota, St. Paul, MN, USA; 4 Department of Animal Science, Michigan State University, East Lansing, MI, USA

## Abstract

**Background:**

*Mycobacterium avium *subsp. *paratuberculosis *(*MAP*), the causative agent of Johne's disease (JD) persistently infects and survives within the host macrophages. While it is established that substantial genotypic variation exists among *MAP*, evidence for the correlates that associate specific *MAP *genotypes with clinical or sub-clinical disease phenotypes is presently unknown. Thus we studied strain differences in intracellular *MAP *survival and host responses in a bovine monocyte derived macrophage (MDM) system.

**Results:**

Intracellular survival studies showed that a bovine *MAP *isolate (B1018) and a human *MAP *isolate (Hu6) persisted in relatively higher numbers when compared with a sheep *MAP *isolate (S7565) at 24-hr, 48-hr and 96-hr post infection (PI). MDMs stimulated with B1018 up-regulated IL-10 at the transcript level and down-regulated TNFα at the protein and transcript levels compared with stimulations by the S7565 and Hu6. MDMs infected with Hu6 showed a down regulatory pattern of IL-10 and TNFα compared to stimulations by S7565. Cells stimulated with B1018 and Hu6 had low levels of matrix metalloprotease-3 (MMP3) and high levels of tissue inhibitor of metalloprotease-1 (TIMP1) at 96-hr PI relative to MDMs stimulated by S7565.

**Conclusion:**

Taken together, results suggest that the bovine (B1018) and the human (Hu6) *MAP *isolates lead to anti-inflammatory and anti-invasive pathways in the macrophage environment whereas the sheep (S7565) *MAP *isolate induces a pro-inflammatory pathway. Thus the infecting strain genotype may play a role in polarizing the host immune responses and dictate the clinicopathological outcomes in this economically important disease.

## Background

Johne's disease (JD) is caused by the intracellular pathogen, *Mycobacterium avium *subsp. *paratuberculosis *(*MAP*). Several molecular techniques have been applied to differentiate and characterize *MAP *isolates from diverse hosts and geographic locations [1-3]. A recent study applied highly discriminatory molecular markers termed short sequence repeats (SSR) to analyze the diversity among *MAP *isolates from a variety of hosts [4]. The results provided evidence for interspecies transmission of several *MAP *genotypes with some showing host specificity. Intriguingly, all genotyping studies addressing diversity using a variety of methods show that *MAP *isolated from human Crohn's disease cases are a subset of *MAP *genotypes widespread in distribution among animal populations. These findings raise several questions regarding the association of specific genotypes with human disease and/or chronic sub-clinical versus overt clinical disease in animals. Since no information on disease phenotypes was obtained when the isolates were acquired for genotyping in our laboratory, logical interpretation of genotype-phenotype associations was not possible. In the absence of clinical data associated with genotypes, and a suitable animal model to rapidly identify strain-associated differences, studying *MAP *interactions in a cellular (macrophage) interphase provides an indirect tool to help dissect the early molecular events that occur during host-pathogen interactions.

Despite the fact that hosts have only a limited number of pathways in which they respond to pathogens, macrophages show both pathogen-specific gene expression profiles and a shared gene expression pattern when infected with diverse bacterial pathogens [5]. An in vitro study of human macrophage responses to a repertoire of genotypically and epidemiologically well defined clinical isolates of *Mycobacterium tuberculosis *(*MTB*) showed a strain dependent host response [6]. A more recent study has shown a shared and a unique gene expression signature by human macrophages stimulated with four isolates of *M. avium *that varied in growth characteristics [7]. Significant differences in cytokine-chemokine profiles or global gene expression profiles in either well-established cell lines (THP-1 or U937) or peripheral blood mononuclear cells (PBMCs) in response to diverse pathogenic and non pathogenic mycobacteria, have also been documented by several recent studies [8-11]. Taken together, available data in the current literature strongly suggests that macrophages infected with mycobacteria have differential gene expression profiles and that the infecting genotypes may dictate down stream host responses. Surprisingly, there have been no reports so far about comparative analyses of diverse clinical isolates of *MAP *within a host/host macrophage, which is a well established fact in other mycobacteria. We believe that this crucial piece of evidence is important in order to understand complex mechanisms underlying the virulence of this economically important pathogen.

Towards these long term goals, in this study we asked if genotypically distinct strains of *MAP *derived from different host species elicit differential responses in bovine monocyte derived macrophages. To test our hypothesis that there would be no strain-specific variation in host responses, we studied the growth kinetics of genotypically distinct strains of *MAP *in both BOMAC cells [12] and bovine monocyte derived macrophages (MDM). We compared the modulation of cytokines such as IL-10, TNFα and matrix metalloproteinase (MMP) genes such as MMP3 and MMP9 as a function of infecting genotype. The importance of these cytokines in JD has been reported elsewhere [13,14]. Cytokines IL-10 and TNFα were evaluated because the relative balance in expression of these cytokines indicates macrophage activation. PBMCs isolated from cattle infected with JD have been shown to up regulate MMP9 and TIMP after stimulation with *MAP *[15]. MMPs when secreted in lower quantities help in leukocyte migration but when secreted in larger quantities cause tissue destruction [16]. A balance between MMP and TIMP is important in the extent of tissue degradation [16]. In summary, in this study we report a differential response of bovine monocyte derived macrophages to a variety of *MAP *isolates.

## Results

### Intracellular survival kinetics of *MAP *strains

**Figure 1:**
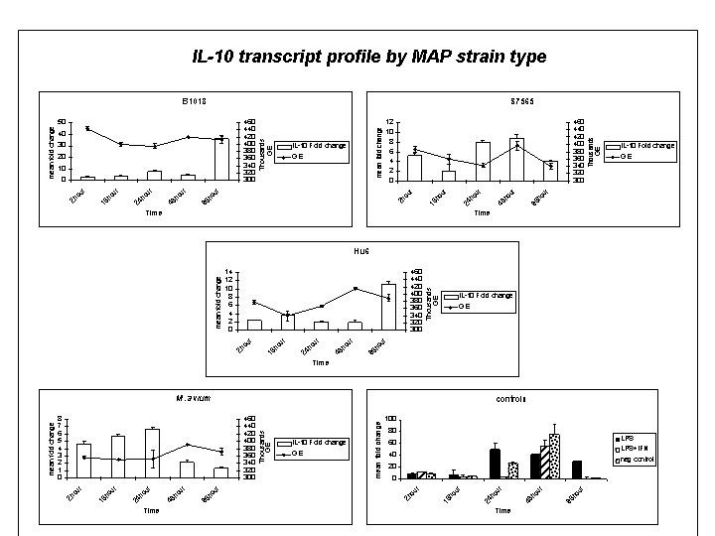
IL-10 mRNA expressed by MDM cells exposed to *M. paratuberculosis *strains over time was measured by Real time RT PCR and fold change in gene expression relative to β actin was calculated by 2^-ΔΔCT ^method. Mean fold change in gene expression is plotted on Y-axis (note that the Y1-axis scales have been optimized for magnitude of IL-10 expression for each strain). Intracellular bacterial numbers based on the amplification of *hsp65 *were calculated based on genome size of *MAP *and represented as genome equivalents (GE) on second Y-axis. MDMs stimulated with B1018 and Hu6 *MAP *isolates increased IL-10 mRNA by 96 hrs PI relative to cell stimulations by S7565 *MAP *isolate and *M. avium*.

**Figure 2:**
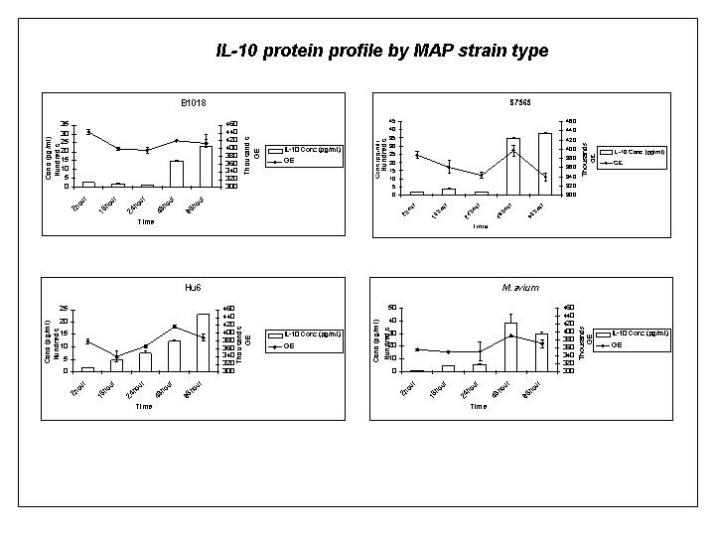
IL-10 protein secreted by MDM cells exposed to *MAP *over time was measured by ELISA. Total amount of protein (pg/ml) is plotted on Y-axis (note that the Y1-axis scales have been optimized for magnitude of IL-10 expression for each strain). Intracellular bacterial numbers were calculated based on genome size of *MAP *and represented as genome equivalents (GE) on second Y-axis. MDMs stimulated with B1018 and Hu6 *MAP *isolates gradually up regulated IL-10 secretion from 2-hr until 96-hr PI.

**Figure 3:**
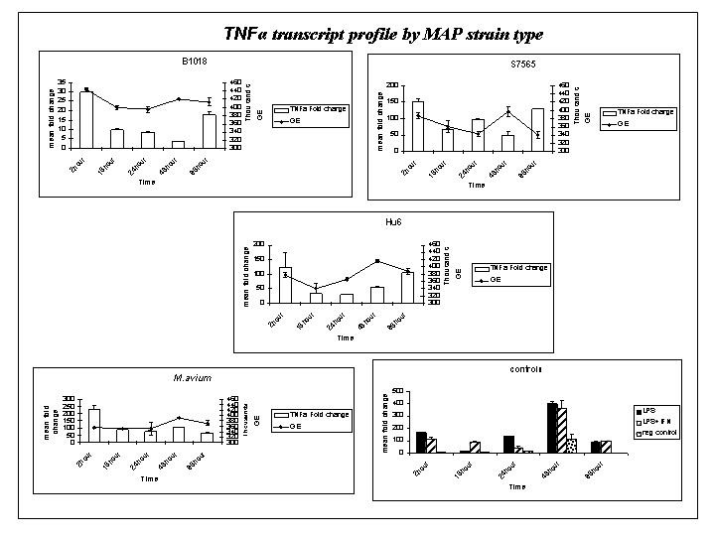
TNFα mRNA expressed by MDM cells exposed to *MAP *over time was measured by Real time RT PCR and fold change in gene expression relative to β actin was calculated by 2^-ΔΔCT ^method. Mean fold change in gene expression is plotted on Y-axis (note that the Y1-axis scales have been optimized for magnitude of IL-10 expression for each strain). Intracellular bacterial numbers based on the amplification of *hsp65 *were calculated based on the genome size of *MAP *and represented as genome equivalents (GE) on second Y-axis. MDMs stimulated with B1018 expressed lower levels of TNFα mRNA relative to other cell stimulations.

**Figure 4:**
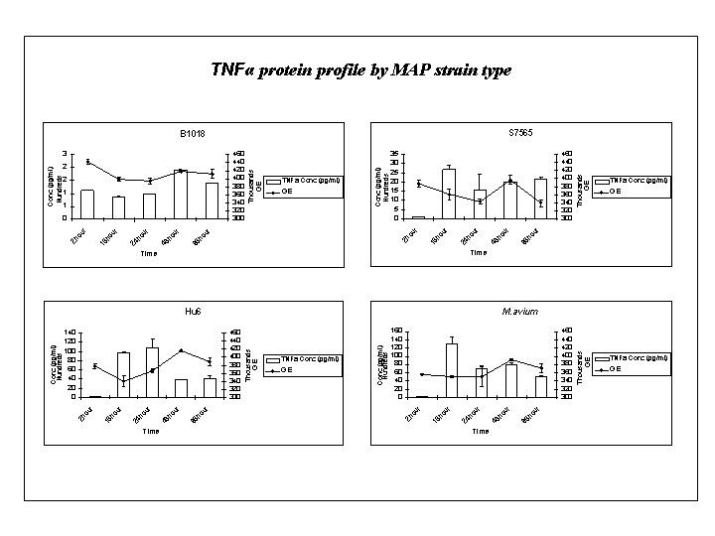
TNFα protein secreted by MDM cells exposed to *MAP *over time was measured by ELISA. Total amount of protein (pg/ml) is plotted on Y-axis (note that the Y1-axis scales have been optimized for magnitude of TNFα expression for each strain). Intracellular bacterial numbers were calculated based on the genome size of *MAP *and represented as genome equivalents (GE) on second Y-axis. MDMs stimulated with B1018 secreted low amounts of TNFα protein relative to other cell stimulations.

B1018 (bovine *MAP *isolate) was efficiently phagocytosed by bovine MDMs and persisted at fairly high numbers when compared to other isolates at all time points (Figures 1, 2, 3, 4). S7565 (sheep *MAP *isolate) decreased in bacterial numbers until 24-hr PI, started to multiply by 48-hr PI and dropped in total intracellular bacterial numbers by 96-hr PI. Hu6 (human *MAP *isolate) declined in numbers until 16-hr post infection and began replicating until 48-hr after infection and started to drop until 96-hr PI. *M. avium *showed a persistent trend until 24 hrs after infection of MDMs and replicated thereafter, until 48 hrs post infection.

In BOMAC cells S7565 multiplied more rapidly and stayed in higher numbers at 96 hr PI relative to B1018. However, in bovine MDM cells B1018 was efficiently internalized and stayed in higher numbers relative to S7565 and Hu6 *MAP *isolates (data not shown).

### IL-10 expression profile

As intracellular bacteria began replication (2–96 hr PI), MDMs stimulated with bovine (B1018) and human (Hu6) *MAP *isolates up-regulated IL-10 mRNA (fig 1) and protein (fig 2) levels over the entire infection period and this peaked from 48-hr to 96-hr PI. There was a positive correlation between IL-10 mRNA gene expression and IL-10 protein secretion (data not shown) observed in the cells stimulated with B1018 and Hu6. Cells stimulated with S7565 and *M. avium *down regulated IL-10 mRNA by 96-hr PI (fig 1). Cells stimulated with *M. avium *showed a gradual increase in IL-10 protein secretion until 48-hr PI and started to drop until 96-hr PI (fig 2). Interestingly, cells stimulated with S7565 showed a sustained increase in IL-10 protein secretion until 96-hr PI (fig 2).

### TNFα expression profile

MDMs stimulated with B1018 and Hu6 isolates showed a down regulatory trend in TNFα mRNA expression from 2-hr to 48-hr PI that switched course to an up-regulatory trend from 48-hr until 96-hr PI (fig 3). Cells stimulated with B1018 gradually up-regulated TNFα protein secretion from 2-hr until 48-hr PI that dropped thereafter (fig 4). Cells stimulated with Hu6 increased TNFα protein secretion until 24-hr PI that showed a down-regulatory trend until 96-hr PI (fig 4). Cells stimulated with S7565 up-regulated TNFα mRNA and protein levels by 96-hr PI (fig 3 &4). At 96-hr PI, the magnitude of TNFα mRNA and protein level was significantly lower (*P *< 0.05) in cells stimulated with B1018 relative to cell stimulations by S7565, Hu6 and *M. avium *(fig 3). Although, cells stimulated with *M. avium *down regulated TNFα protein secretion from 16-hr until 96-hr PI, the amount of protein detected was significantly higher (*P *< 0.05) relative to other stimulations (fig 4).

### MMP3 mRNA expression

MDMs stimulated with B1018 increased the production of MMP3 mRNA at 16-hr which was followed by a rapid decline until 96 hrs PI (table 1). Cells stimulated with S7565 produced a peak level of MMP3 at 24-hr that declined by 96-hr. While MMP3 mRNA production by cells stimulated with B1018 was high at 16-hr relative to cells stimulated by S7565 and Hu6, the data was not statistically significant (*P *= 1.0). At 24-hr there was a significant increase (*P *< 0.05) in the production of MMP3 mRNA by cells stimulated with S7565 relative to other stimulations. There was a gradual up-regulation of MMP3 mRNA production by MDMs stimulated with Hu6 until 48 hrs and declined by 96-hr.

### MMP9 mRNA expression

Cells stimulated with B1018 showed lower levels of MMP9 mRNA production at 48-hr and 96-hr PI relative to cell stimulations with other *MAP *isolates (table 2). Cells stimulated with S7565 had a peak MMP9 mRNA production at 48-hr after infection, which was significantly higher (*P *< 0.05) when compared with stimulations by other *MAP *isolates (table 2). Cells stimulated with Hu6 also showed an up-regulatory trend in MMP9 mRNA production until 96-hr (table 2). Cells stimulated with Hu6 had significantly (P < 0.05) low MMP9 mRNA levels relative to cells stimulated with S7565 at 24-hr and 48-hr PI (table 2).

### TIMP1 mRNA expression

LSMean values of TIMP1 mRNA levels suggested that MDMs stimulated with B1018 showed higher levels relative to cell stimulations by S7565 (data not statistically significant) (*p *= 1.0). There was a peak production of TIMP1 mRNA observed at 96-hr in MDMs stimulated with B1018 (table 3).

## Discussion

Macrophages are the first line of host defense against any invading bacteria. Despite the fact that macrophages offer a hostile environment to several pathogenic bacteria, *MAP *is able to persistently survive and replicate within the phagosome environment of host macrophages. Studying the biochemical processes operating at the host-pathogen interface will help elucidate the mechanisms by which *MAP *has developed expertise to survive and replicate inside macrophages. Over the years many researchers have employed techniques such as microarray, semi-quantitative PCR, Q-RT-PCR to study the gene expression profiles in a cellular model after infection with pathogenic mycobacteria including type strains of *MAP *[9,17,18]. However, a possible *MAP *genotype-disease phenotype association has not been established despite the evidence that there is diversity in the genotypes of *MAP *strains isolated from several different hosts [19,20].

In the absence of a well characterized experimental animal model to study host pathogen interactions of *MAP *in JD, cellular models have served as a helpful surrogate to researchers [21-24]. While BOMAC cells provide an easy to use immortalized cell line to study host-pathogen interactions, our studies with this cell line support earlier observations by Sager *et al*. [25] further highlighting that this bovine monocytoid cell line may differ in their behavior compared with MDMs. Thus, we chose to characterize *MAP *strain dependent host response only in bovine MDM cells.

Bovine monocyte derived macrophages infected with *MAP *have been previously employed to study changes in cytokine profiles [21,26]. In the present study we have demonstrated a *MAP *genotype dependent phenotype characterized by differences in the cytokine profiles such as IL-10, TNFα and MMPs in MDMs. The importance of IL-10 and TNFα in JD has been reported elsewhere [13,14]. IL-10 inhibits macrophage activation and is one of the major anti inflammatory cytokines [27]. TNFα is a major inflammatory cytokine produced by activated macrophages and is involved in controlling bacterial replication [28-30].

Our results based on the data generated using MDM cells obtained from two different animals consistently showed that B1018 (bovine *MAP *isolate) efficiently entered and remained in higher numbers within MDM cells relative to other *MAP *isolates. Cells stimulated with B1018 up-regulated expression of IL-10 mRNA (*P *< 0.005) while expression of TNFα mRNA was down-regulated relative to other *MAP *isolates. This was also evident in the relatively low proteins identified in the culture supernatant. Previous studies also reported a similar phenotype for the ATCC 19698 strain of *MAP *in bovine macrophages [21]. Khaleifh et. al [31] reported an up regulatory pattern of IL-10 secretion in bovine macrophages following infection with a type strain of *MAP *which is consistent with our findings, although the magnitude of up regulation at the protein level was much lower compared with their IL-10 levels. When compared to B1018, Hu6 and S7565 strains significantly down regulated IL-10 mRNA and up regulated TNFα mRNA. MDMs stimulated with S7565 and Hu6 *MAP *strains had significantly high amounts (P < 0.05) of TNFα and IL-10 secreted into culture supernatants relative to B1018 strain. A similar proinflammatory response by dendritic cells to whole cell *MAP *(strain 316F) infection has been recently demonstrated [32]. The study also documented that stimulation of dendritic cells by a recombinant immunodominant antigen of *MAP *included severe anti-inflammatory responses invoking the hypothesis that the differential in macrophage responses seen in our study may have occurred due to differences in expression of specific virulence genes by the strains studied. Our studies with SSR analysis [4,19,33,34] and SNP analysis (Zhu and Sreevatsan, unpublished) suggest that specific genotypes may be associated with subclinical disease while others may lead to clinically overt disease. Additionally, in vitro analysis of *MAP *survival within primary macrophage cells in the present study show clear distinction in entry, survival and persistence as a function of genotypes. DNA microarray analysis of the genome content of *MAP *isolates employed in this study using MAA104 array revealed that several large sequence polymorphisms (LSPs) were missing in S7565 when compared to B1018 (Semret M, presented at 8^th ^International Colloquium on Paratuberculosis). While this may explain the variations between sheep (S7565) and bovine (B1018) isolates, the variations in host response to bovine (B1018) or human (Hu6) genotypes of *MAP *may exist in SNPs and/or variations in bacterial gene regulation within the host. Confirmation of this finding will require analysis of a larger genotypically diverse collection for both host and pathogen gene expression. Comparisons of infections with a type strain of *MAP *and *M. avium avium *in bovine MDMs have revealed an increased expression of TNFα in cells infected with *M. avium avium *and a down regulation of IL-10 [21]. The *M. avium intracellulare *strain employed in our study showed similar trends in TNFα production when compared to other *MAP *strains studied. However, an increased IL-10 level was detected in culture supernatants infected with *M. avium intracellulare *compared to other strains at 96-hr PI. The differences observed could be because the *M. avium intracellulare *strain utilized in this study was unique in that this strain carried IS*900*, an insertion element that was once considered unique to *MAP*.

Matrix metalloproteinases (MMPs) are a family of calcium-dependent proteinases [35] involved in cell migration, tissue remodeling and destruction. Tissue inhibitors of MMP (TIMP) inhibit the activity of MMP. A balance between MMP and TIMP produced at the site of inflammation influences the ability of immune cells to migrate and the amount of tissue destruction caused [36]. PBMCs isolated from cattle infected with JD have been documented to up-regulate MMP9 and TIMP after stimulation with *MAP *[15,37]. MMP and TIMP are reported to play a functional role in infections caused by pathogenic mycobacteria [38]. In our study, cells stimulated with B1018 down-regulated MMP9 while up-regulating TIMP1 production relative to MDMs stimulated with Hu6 and S7565 strains. This is consistent with the idea that these *MAP *genotypes (finger print: 7G4GGT) lead to an anti-inflammatory and anti-invasive milieu allowing their persistence and survival within macrophages. THP-1 cells infected with *MTB *have shown significant production of MMP9 and TIMP1 but not MMP1 [16]. MMP9 when secreted at low levels aid leukocyte migration to sites of inflammation. However, in large amounts, MMP9 causes tissue destruction [16]. It has also been shown that TNFα production at the site of inflammation correlates with MMP9 production [39]. Our observations are consistent with the idea that S7565 strain may elicit a relatively more invasive pathway during infection. Taken together, our studies demonstrate a *MAP *genotype-dependent response in a bovine monocyte derived macrophage model.

## Conclusion

The present findings provide key insights into the *MAP *genotype-disease phenotype associations. Further analysis of this complex "ancient dialogue" between *MAP *and macrophages derived from its natural host (bovines) will help elucidate the pathogenesis associated with different genotypes isolated from diverse host species. These studies will aid in understanding the proximal events involved in the progression of JD and the virulence of *MAP *isolates thus enabling design of early intervention strategies. Future studies with a broader range of *MAP *isolates with common and unique genotypes associated with JD are warranted.

## Methods

### Preparation of monocyte derived macrophages (MDM)

Two colostrum-deprived Holstein bull calves obtained from a Johne's disease free herd served as a source for peripheral blood and MDMs. The calves were tested 4 and 8 weeks after birth by fecal culture and serum ELISA and were confirmed to be JD free. The protocol used for the preparation of MDMs is described elsewhere [40]. Briefly, peripheral blood was drawn from jugular vein into acid-citrate dextrose containing vacutainers (BD Vacutainer, Rutherford, NJ). Blood was centrifuged at 2000 rpm for 20 minutes to obtain a clean buffy coat. Buffy coats were re-suspended in sterile 1× PBS (1:10 dilution) and overlaid on Histopaque (Sigma Aldrich, St. Louis, Mo) following manufacturer's recommendations. The tubes were centrifuged at 400 × g for 30 minutes at room temperature to separate mononuclear cells from other polymorphonuclear leukocytes. Mononuclear cells from the interphase were harvested carefully using a sterile Pasteur pipette and transferred to a second sterile 50-ml conical tube. The cells were washed with 10-ml of 1× PBS at 250 × g for 10 minutes. Supernatant was discarded and the cell pellet was re-suspended in a small volume of 1× PBS. The mononuclear cells were transferred to TEFLON jars (Savillex Corporation, Minnetonka, MN) containing RPMI 1640 medium supplemented with L-glutamine, HEPES and 20% autologous serum. The jars were incubated at 37°C, 95% air and 5% CO_2 _for 4 days. After four days monocytes differentiated and became larger in size. Differentiated monocytes were counted using a hemacytometer and seeded onto the tissue culture plates at appropriate dilutions and incubated at 37°C, 95% air and 5% CO_2 _for 2 hours. Plates were washed twice with sterile 1× PBS to remove the non-adherent cells. The adherent cells were used for all in-vitro infections.

### *M. paratuberculosis *isolates

The selected *MAP *strains included B1018 (SSR fingerprint: 7G4GGT9nG), S7565 (SSR fingerprint: 15G3GGT), Hu6 (SSR fingerprint: 7G5GGT11nG) and Ma6043 (IS*900 *positive isolate identified as *M. avium intracellulare *by multiple target analyses; no SSR data) (11). B1018 was isolated from a cow with clinical JD and carried a fingerprint which is common to about 12% of bovine *M. paratuberculosis *strains in a national collection (Harris and Sreevatsan, Unpublished) and in greater than 45% of isolates from Ohio (21), S7565 was isolated from sheep and Hu6 was isolated from a Crohn's disease patient.

### Bacterial cultures

All the *MAP *cultures were incubated at 37°C on MB7H9 plates supplemented with OADC enrichment medium and Mycobactin J. After 3–4 weeks of growth on MB7H9 plates, cultures were confirmed to be free of other contaminating organisms as determined by nil growth on BHI or blood agar plates incubated overnight at 37°C. Few colonies from MB7H9 plate cultures were inoculated into MB7H9 broth culture supplemented with OADC enrichment medium and Mycobactin J and incubated at 37°C for 3 days to achieve actively growing *MAP*. Three day-old cultures were used to obtain an optical density at 600 nm (OD_600_) to determine the colony forming units (cfu) of bacteria using the formula: 0.3 at OD600 = 10^9^cfu/ml, and applied in all in vitro infections of bovine MDM. Bacteria were used at a 5:1 multiplicity of infection.

### Experimental design

MDM monolayers were grown on six well tissue culture plates. Cells were stimulated with three *MAP *isolates (B1018: bovine, S7565: sheep, and Hu6: human) and one IS*900 *positive non-*MAP *isolate (6043: identified as *M. avium intracellulare*). Lipopolysachharide (LPS; 100 μg/ml) (Sigma Aldrich, St. Louis, Mo) stimulated and LPS (100 μg/ml) in conjunction with recombinant bovine IFNγ (14 ng/ml) (Serotec, Raleigh, NC) stimulated cells served as positive controls where as, nil stimulated MDM served as negative controls in the experiment. All the stimulations were carried out in triplicates and were performed simultaneously. Cell stimulations were repeated twice on MDMs from each animal to evaluate consistency in the data generated. The culture plates were incubated at 37°C, 95% air and 5% CO_2 _until used. At each time point (2-hr, 16-hr, 24-hr, 48-hr and 96-hr) cells and culture supernatants from all the treatments were harvested. The culture supernatant was collected and stored at -70°C until used for ELISA. Monolayers were immediately washed twice with sterile, pyrogen-free 1× PBS and used in RNA extractions.

### RNA extraction and real time Q-RT-PCR

RNA extractions were carried out using TRIzol reagent (Invitrogen, Carlsbad, CA) following manufacturers' recommendations. All the RNA samples were treated with RNAse free DNAse I (Ambion, Austin, TX) according to manufacturer's recommendations and stored at -70°C until utilized in QRT-PCR. Prior to their use, RNA was assessed for the quality and quantity using a spectrophotometer (GeneQuant pro by Amersham Bio sciences Corp, Piscataway, NJ). Subsequently, all the samples were diluted using nuclease free water at a concentration of 10 ng/μl. Later, Real time Q-RT-PCR was performed using Light Cycler system (Roche Diagnostics, Indianapolis, IN). Single step RT PCR was performed using Quantitect SYBR Green RT PCR kit (Qiagen Inc., Valencia, CA). Briefly, each reaction mixture contained 10 μl of master mix, 0.2 μl of RT mix, 2 μl of template RNA and gene specific (IL-10, TNFα, MMP3, MMP9, or TIMP1) primers. Reactions were performed in 20 μl light cycler capillaries (Roche, Indianapolis, IN). Primers used to analyze all the transcripts have been reported else where. The Q-RT-PCR data was analyzed by using 2^-ΔΔCT ^method as previously described [41,42].

### Survival analysis of *MAP *strains

MDMs or BOMAC cells [12] were infected with three *MAP *isolates and an *M. avium *at an MOI of 1:5 as described above. All the stimulations were carried in triplicates and were performed simultaneously. Infected cells were incubated at 37C, 5%CO_2 _until desired. At each time point (0, 2, 16, 24, 48 and 96 hrs post infection) culture plates were removed and monolayers were vigorously washed for three times with 1× PBS to get rid of loosely adherent cells and external bacteria. DNA from the monolayers was extracted using TRIzol (Invitrogen, Carlsbad, CA) following manufacturer's recommendations.

Briefly, Monolayers were lysed on culture plates using 1 ml of Trizol reagent. Samples were then mixed with zirconium beads (0.1 mm) and homogenized for 3 minutes using mini bead beater (Biospec products, Bartlesville, OK). This effectively released mycobacterial DNA. Later, samples were mixed with 200 μl of chloroform and centrifuged at 1500 rpm for 5-min at 4C. Pellet was washed twice with 0.1 M sodium citrate in 10% ethanol. Finally pellet was washed with 75% ethanol, air dried and dissolved in sterile distilled water and stored at -20C until used. DNA was used in the real time PCR to quantitate *hsp65 *gene of *MAP*. Since *hsp65 *is a single copy gene, it provided a good estimate of the total number of organisms that survived macrophage infection over time. All the amplifications were carried out in Light cycler (Roche, Indianapolis, IN) using quantitect SYBR Green (Qiagen, Valencia, CA).

Briefly, each reaction mixture contained 10 μl of master mix, 2 μl of template DNA and gene specific primers (forward – 5' GCC GCT GCT GAT CAT CGC CGA 3') (reverse – 5' CCT TGG TGA CGA CCT T 3'). Reactions were performed in 20 μl light cycler capillaries (Roche, Indianapolis, IN). Obtained ct (crossing time points) values from the real time PCR were converted to genome equivalents. Based on the genome size of *MAP *one genome equivalent was calculated to be equal to about 9.9 fmg of *MAP *DNA.

### Development of *hsp65 *standard for quantification purposes

DNA extracted from the broth cultures of *MAP *was pooled and the concentrations were determined. Five, ten-fold dilutions of the DNA was made and used as template in the real time PCR for amplification of *hsp65 *gene. Obtained ct values were imported onto an Excel spread sheet. Ct vales were plotted against genome equivalents (Y axis) and regression analysis was performed. This regression equation was used to estimate the bacterial numbers (as genome equivalents) in all treatment samples.

### Quantitation of extracellular cytokine production by ELISA

Cytokine sandwich ELISA was used to detect IL-10 and TNFα from the culture supernatants. The protocol was adopted and modified to our conditions as described [43,44]. Briefly, 96 well micro titer plates (NUNC, Rochester, NY) were coated with mouse anti bovine IL-10 (Serotec Inc, Raleigh, NC) or mouse anti bovine TNFα (generous gift from Dr. Paape, USDA, Ames, IA) for overnight at 4°C. Next day plates were washed with PBS Tween20 (0.05%) and blocked with PBS/BSA (0.5%) for one hour at room temperatures. Culture supernatants were added to the plates. The standard protein was serially diluted and added to the corner wells. Standard protein for TNFα was purchased from Endogen where as standard protein for IL-10 was a generous gift from Dr. Howard, Animal Research Center, UK. The plates were incubated at 4°C overnight. The plates were then washed briefly with PBS Tween20 (0.05%) and mouse anti bovine IL-10 labeled with biotin (Serotec Inc, Raleigh, NC) or rabbit anti 1 bovine TNFα (generous gift from Dr. Paape, USDA, Ames, IA) was added and incubated at 37°C for 3 hours. Plates were then washed and streptavidin HRP (Serotec Inc, Raleigh, NC) was added to plates that were used for IL-10 detection, incubated for an hour at room temperature. Anti rabbit IgG1 labeled with HRP (Biorad, CA) was added to plates for detecting TNFα and incubated at room temperature for 2 hours. All the plates were then added with color developing solution (Biorad, CA) and plates were read using ELISA plate reader at 413 nm.

### Estimation of protein concentration of IL-10 standard protein

The standard protein used for quantitating IL-10 in culture supernatants was gifted by Dr. Howard, UK. The source of recombinant bovine IL-10 was culture supernatant obtained from Cos-7 cells transfected with a plasmid encoding bovine IL-10. The IL-10 supplied had 3000 biological units per ml. One biological unit of IL-10 supplied to us corresponded to an equivalent of 600 ng of protein.

## Authors' contributions

Dr. Janagama, performed cell infections, and analyzed the data. Dr. Jeong, helped in the design and analysis of ELISAs for cytokines studied. He also provided intellectual input during cell-infection study design. Dr. Kapur provided intellectual help during the study design and data analysis. Dr. Coussens was responsible for the original concept development with Dr. Sreevatsan. He also helped in the performance of MAP survival studies. Dr. Sreevatsan developed the concepts, designed MAP infection studies, and analyzed the data with Dr. Janagama. Dr. Sreevatsan also helped prepare the manuscript for consideration of publication.

## Figures and Tables

**Table 1:** LSMean values with Standard errors of the MMP3 gene transcripts. Depicts the LSMean values with Standard errors of the MMP3 gene transcripts expressed by macrophages over time and measured by Real Time RT PCR as described in materials and methods. Values with same superscript (a, b, c, p, q, r) indicate significant differences among the strains at that time point PI.

Strain	Time post infection				
	2 hour	16 hour	24 hour	48 hour	96 hour

B1018	44.3 ± 4.77	574.6 ± 315^a^	259.4 ± 50.5^p^	121.6 ± 26	72.3 ± 24.3
S7565	55.6 ± 5.17	25.7 ± 6.6^b^	10057 ± 2104^p,q,r^	5451 ± 453	406 ± 17.9
Hu6	5.4 ± 0.2	25 ± 3.9^c^	134 ± 19.6^r^	319 ± 6.2	61.2 ± 10.7
*M. avium*	22.3 ± 2.2	6794 ± 133^a,b,c^	846 ± 107^q^	166.7 ± 22.8	149 ± 3.6
PC1^x^	56.6 ± 8.5	555 ± 51.6	26180 ± 4595	16742 ± 2938	1840 ± 678
PC2^y^	188.8 ± 39	15825 ± 2625	8164 ± 160	35563 ± 3307	554 ± 8.1
NC^z^	14.8 ± 7.5	43.9 ± 3.6	398 ± 29.4	2629 ± 77.3	1.7 ± 0.18

^x ^indicates positive control 1 (PC1) – cells stimulated with LPS

^y ^indicates positive control 2 (PC2) – cells stimulated with LPS in conjunction with recombinant bovine Interferon gamma

^z ^indicates negative control (NC) – unstimulated cells

**Table 2:** LSMean values with Standard errors of the MMP9 gene transcripts. Depicts the LSMean values with Standard errors of the MMP9 gene transcripts expressed by macrophages over time and measured by Real Time RT PCR as described in materials and methods. Values with same superscript (a, b, c, d) indicate significant differences among the strains at that time point PI.

Strain	Time post infection				
	2 hour	16 hour	24 hour	48 hour	96 hour

B1018	3 ± 0.47	18.2 ± 0.81	82.7 ± 19.2	109.8 ± 12.3^c^	66.6 ± 10^d^
S7565	1.4 ± 0.09	0.07 ± 0.03	130.8 ± 8.3^a^	760.3 ± 26^c^	117.4 ± 6.3
Hu6	4.2 ± 0.72	10.9 ± 0.64	45.9 ± 2.25^a,b^	321.3 ± 29.8	629 ± 49.2
*M. avium*	0.94 ± 0.27	53.2 ± 1.3	131.3 ± 10.9^b^	318.5 ± 38.9	220.6 ± 9.7^d^
PC1^x^	0.7 ± 0.06	49.4 ± 3.1	648 ± 9.5	365.8 ± 8.9	19.8 ± 1.8
PC2^y^	0.44 ± 0.01	65 ± 2.2	294 ± 10	1564 ± 91.9	5.4 ± 2.2
NC^z^	0.3 ± 0.07	2.07 ± 0.2	5.6 ± 2.7	0.1 ± 0.01	6.7 ± 0.23

^x ^indicates positive control 1 (PC1) – cells stimulated with LPS

^y ^indicates positive control 2 (PC2) – cells stimulated with LPS in conjunction with recombinant bovine Interferon gamma

^z ^indicates negative control (NC) – unstimulated cells

**Table 3:** LSMean values with Standard errors of the TIMP1 gene transcripts. Depicts the LSMean values with Standard errors of the TIMP1 gene transcripts expressed by macrophages over time and measured by Real Time RT PCR as described in materials and methods.

Strain	Time post infection				
	2 hour	16 hour	24 hour	48 hour	96 hour

B1018	6.45 ± 0.28	16.65 ± 8.57	6.2 ± 0.09	11.63 ± 1.42	45.9 ± 8.74
S7565	12.4 ± 1.88	2 ± 0.67	5.74 ± 1.9	9.26 ± 0.5	25.2 ± 0.25
Hu6	3.12 ± 0.79	0.87 ± 0.16	1.1 ± 0.34	2.3 ± 0.3	34.6 ± 5.7
*M. avium*	2.62 ± 0.18	3.2 ± 1.5	3.5 ± 0.07	4.2 ± 0.36	6.4 ± 1.25
PC1^x^	6.1 ± 0.6	3.8 ± 0.04	10.7 ± 1.52	36.4 ± 3.39	159.7 ± 1.57
PC2^y^	6.3 ± 0.34	24 ± 1.53	2.2 ± 0.65	52.3 ± 6.91	38.6 ± 5.1
NC^z^	6.2 ± 0.91	26.6 ± 3.91	49.8 ± 23.26	584.2 ± 71.4	8.1 ± 3.46

^x ^indicates positive control 1 (PC1) – cells stimulated with LPS

^y ^indicates positive control 2 (PC2) – cells stimulated with LPS in conjunction with recombinant bovine Interferon gamma

^z ^indicates negative control (NC) – unstimulated cells
